# Terbium Functionalized Schizochytrium-Derived Carbon Dots for Ratiometric Fluorescence Determination of the Anthrax Biomarker

**DOI:** 10.3390/nano9091234

**Published:** 2019-08-30

**Authors:** Lina Zhang, Zhanwei Wang, Jingbo Zhang, Changliang Shi, Xiaoli Sun, Dan Zhao, Baozhong Liu

**Affiliations:** 1 College of Chemistry and Chemical Engineering, Henan Polytechnic University, Jiaozuo 454003, China; 2 Chemical Laboratory, Medical Instrument Testing Institute of Henan province, Zhengzhou 450018, China

**Keywords:** carbon dots, dipicolinic acid, Tb^3+^, schizochytrium, ratiometric fluorescence nanoprobe

## Abstract

Efficient and instant detection of biological threat-agent anthrax is highly desired in the fields of medical care and anti-terrorism. Herein, a new ratiometric fluorescence (FL) nanoprobe was elaborately tailored for the determination of 2,6-dipicolinic acid (DPA), a biomarker of anthrax spores, by grafting terbium ions (Tb^3+^) to the surface of carbon dots (CDs). CDs with blue FL were fabricated by a simple and green method using schizochytrium as precursor and served as an FL reference and a supporting substrate for coordination with Tb^3+^. On account of the absorbance energy transfer emission effect (AETE), green emission peaks of Tb^3+^ in CDs-Tb nanoprobe appeared at 545 nm upon the addition of DPA. Under optimal conditions, good linearity between the ratio FL intensity of *F*_545_/*F*_445_ and the concentrations of DPA was observed within the experimental concentration range of 0.5–6 μM with the detection limit of 35.9 nM, which is superior to several literature studies and significantly lower than the infectious dosage of the *Bacillus anthracis* spores. Moreover, the CDs-Tb nanoprobe could sensitively detect DPA in the lake water sample. This work offers an efficient self-calibrating and background-free method for the determination of DPA.

## 1. Introduction

Anthrax is a well-known disease caused by *Bacillus anthracis*, which can affect almost all warm-blooded animals, including human beings, resulting in deadly infections after inhalation of over 10^4^ spores in 36 h [1]. Since the spores of *Bacillus anthracis* are highly environmentally adaptive, they have been developed as a biological weapon, which makes them a biohazard threat [2]. As a main ingredient of the bacterial spores, 2,6-dipicolinic acid (DPA) represents 5−15% of the dry mass of the spores and can be served as a typical anthrax biomarker [3]. Thus, developing an efficient and accurate method for DPA detection is very important in the fields of medical care and anti-terrorism.

Compared with traditional detection methods for DPA, fluorescence-based sensing methods have attracted plenty of interest owing to their real-time, economic, highly selective, and sensitive features [4,5]. Among these, sensing platforms based on rare earth ions (Ln^3+^) for DPA determination have received considerable attention due to their high coordination ability with DPA and their excellent spectroscopic properties, namely, large stokes shift, sharp emission bands, and long fluorescence (FL) lifetime [6]. When coordinated with DPA, the FL intensity of Ln^3+^ becomes more intense via the absorbance-energy transfer-emission effect (AETE) [7,8]. However, most reported measurements rely on single fluorescent signal changes of Ln^3+^, which may be easily influenced by environmental or instrumental factors [3,6,7]. To conquer this limitation, ratiometric FL probes that contain another FL spectral peak as an internal reference would be an ideal choice to improve the accuracy of the detection. Hitherto, various Ln^3+^-incorporated fluorescent ratiometric platforms for DPA detection have been exploited, such as silicon quantum dots [4], solid films [1], Micelle [9], and metal-organic framework [10].

As a new family member of carbon nanomaterials, carbon dots (CDs) have recently inspired substantial attention because of prominent properties such as facile preparation, low cost, high photostability, and nontoxicity [11,12]. Although a few CDs-based FL nanoprobes for DPA determination have been reported [13,14], further improvement is still needed for the DPA sensors, for example, more facile synthesis, lower cost, and higher photostability and sensitivity. Moreover, it is worth mentioning that using a more environmentally friendly approach to synthesize CDs with fine quality remains a pressing problem waiting to be resolved [15]. Using renewable and low cost green biomass as raw materials to synthesize CDs will inevitably promote the sustainable development of CDs and their applications.

**Scheme 1:**
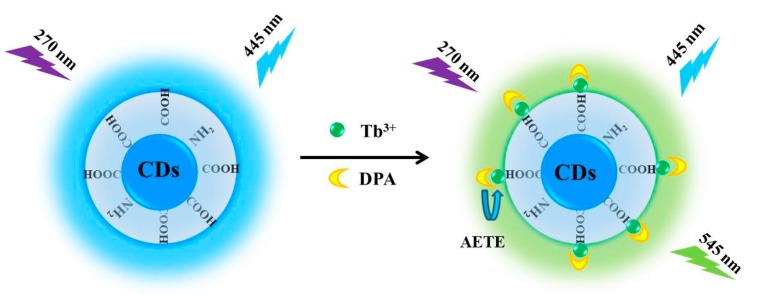
Schematic diagram of the CDs-Tb nanoprobe for DPA recognition.

Herein, CDs with bright blue FL were prepared by a simple and green method using schizochytrium (a kind of microalgae) as precursor. Subsequently, a new ratiometric FL nanoprobe (CDs-Tb) was prepared for the determination of DPA by grafting Tb^3+^ onto the surface of CDs (Scheme 1). Under optimal conditions, good linearity between the ratio FL intensity of *F*_545_/*F*_445_ and the DPA concentrations was observed within the experimental concentration range of 0.5–6 μM with the detection limit of 35.9 nM. Moreover, the CDs-Tb could realize sensitive detection of DPA in lake water samples. The comparison of several existing FL nanoprobes for DPA detection is listed in Table S1, indicating good sensitivity of our sensing system compared with previously reported ones [4,14,16,17,18,19].

To the best of our knowledge, this is the first example of CDs prepared from microalgae and pure water by using a hydrothermal method [15,20]. Although one case of hydrothermal synthesis of microalgae-based carbon dots has been reported, formaldehyde aqueous solution was added during the hydrothermal reaction, which was obviously not environmentally friendly [20]. Moreover, this work offers an efficient self-calibrating and background-free method for the determination of DPA.

## 2. Materials and Methods

### 2.1. Materials

Tris(hydroxymethyl)aminomethane, 2,6-dipicolinic acid (DPA), m-phthalic (mPA), o-phthalic (oPA), benzoic (BA), glutamic (Glu), D-aspartic (Asp) acid, Tb(NO_3_)_3_·6H_2_O and nitrate salts of metal ion of analytic grade were purchased from Shanghai Energy Chemical Corporation (Shanghai, China). Schizochytrium were purchased from Wudi Green Science Engineering Co., Ltd. (Shandong, China). Cellulose dialysis membrane were purchased from Jingke Hongda Biotechnology Co., Ltd. (Beijing, China).

### 2.2. Preparation of CDs

**Scheme 2:**
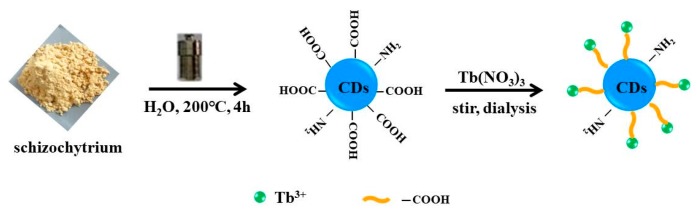
Schematic diagram of the preparation for CDs and CDs-Tb.

CDs with blue FL emissions were fabricated by a green and facile hydrothermal method (Scheme 2). Briefly, 2.0 g schizochytrium and 10 mL distilled water were placed in a Teflon-lined stainless steel vessel (23 mL) and heated at 200 °C for 4 h. The obtained mixture was centrifuged to remove large particle residues. Subsequently, the redundant supernatant was dialyzed for two days via a cellulose dialysis membrane (MWCO 1000) in the pure water. After drying by lyophilization, CDs powders were collected.

### 2.3. Preparation of CDs-Tb

Then, 0.1 mmol Tb(NO_3_)_3_ was added into 10 mL of aqueous CDs (1.0 mg·mL^−1^) solution. At room temperature, the mixtures were stirred for 2 h and then subjected to dialysis. “Free” Tb^3+^ ions were removed via a cellulose membrane (MWCO 500) in pure water for 2 days. After drying by lyophilization, CD-Tb powders were collected.

### 2.4. Characterization

UV-Vis absorption spectra were measured on a Varian UV-Cary100 spectrophotometer (Varian Inc., Palo Alto, CA, USA). Infrared spectrum (IR) was determined on a Pristige IR21 FTIR spectrometer (Shimadzu, Kyoto, Japan). FL spectra were performed on a FluoroMax-P spectrophotometer (Horiba Jobin Yvon, Paris, France). Images of transmission electron microscopy (TEM) were collected by a Tecnai G2 F20 TEM characterization (FEI, Hillsboro, OR, USA). X-ray photoelectron spectra (XPS) were measured on a ESCALAB 250Xi (Thermo fisher Scientific, West Sussex, UK). The lifetime measurements were determined on an Edinburgh fls1000 FL spectrometer (Edinburgh Instruments Ltd, Livingston, UK). The absolute quantum yields were determined using an integrating sphere on a Japan Hamamatsu Quantum Yield Determination System C9920-02G (Hamamatsu Photonics Co., Ltd., Hamamatsu, Japan).

### 2.5. Ratiometric Fluorescence Detection for DPA

Then, 20 μL stock solution of CDs-Tb (1.0 mg·mL^−1^) was mixed with HCl-Tris buffer (pH 7.6) solution; after that, various concentrations of DPA (0, 0.5, 1, 1.5, 2, 2.5, 3, 3.75, 4.5, 6, 7.5, 9 μM; aqueous solution, the concentration of DPA stock solution was 0.5 mM) were added. The total volume of the mixture was 2.0 mL. FL spectra were recorded under the excitation wavelength of 270 nm. In the selectivity detection towards DPA, the concentrations of the selected interferences and the DPA were kept as 10 μM and 4 μM, respectively. The FL titrations and the selectivity detection experiments were carried out three times to achieve reliable results.

To investigate the reaction mechanism between CDs-Tb and DPA, time-resolved decay measurements were carried out. CDs-Tb solutions (0.1 mg·mL^−1^) containing 0 or 80 μM of DPA were tested (pH 7.6). The emission wavelengths were 445 and 545 nm, while the excitation wavelength was 270 nm.

As for analysis in a real sample, a lake water sample taken from our campus was first centrifuged to remove larger insoluble impurities. Then, small insoluble impurities were removed by filtration. Subsequently, 20 μL stock solution of CDs-Tb was mixed with 2.0 mL HCl-Tris butter (pH 7.6) solution, which was prepared by using the above filtered lake water sample. After that, DPA aqueous solutions (1, 2, 5 μM) were added into the above mixture. FL spectra were recorded (λ_ex_ = 270 nm), and each test was repeated three times.

## 3. Results

### 3.1. Characterization of the CDs

**Figure 1:**
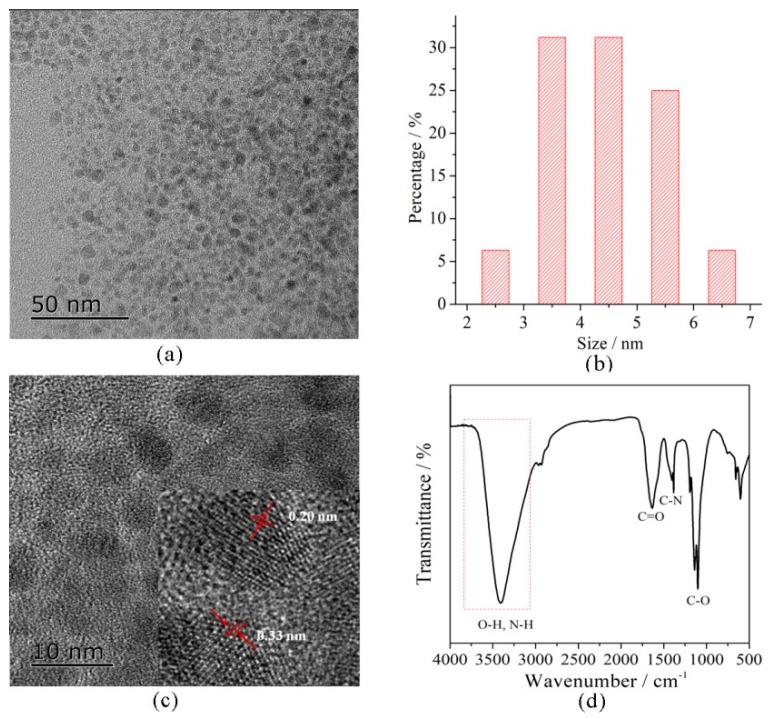
(**a**) TEM image; (**b**) the size distribution histogram; (**c**) HRTEM images; and (**d**) FTIR spectrum of CDs.

The schizochytrium-based CDs were facilely synthesized by a hydrothermal method (Scheme 2). TEM analysis was performed to characterize the microstructure of the CDs. As depicted in Figure 1a, the CDs were spherical shapes and showed monodispersity. The mean diameter of the CDs was 4.5 nm with size distribution from 2.8 to 6.3 nm (Figure 1b). As depicted in Figure 1c, clear lattice fringes of 0.33 nm and 0.20 nm could be observed, which were respectively close to the (002) and the (020) planes of graphitic carbon, implying its graphitic nature of CDs [21].

**Figure 2:**
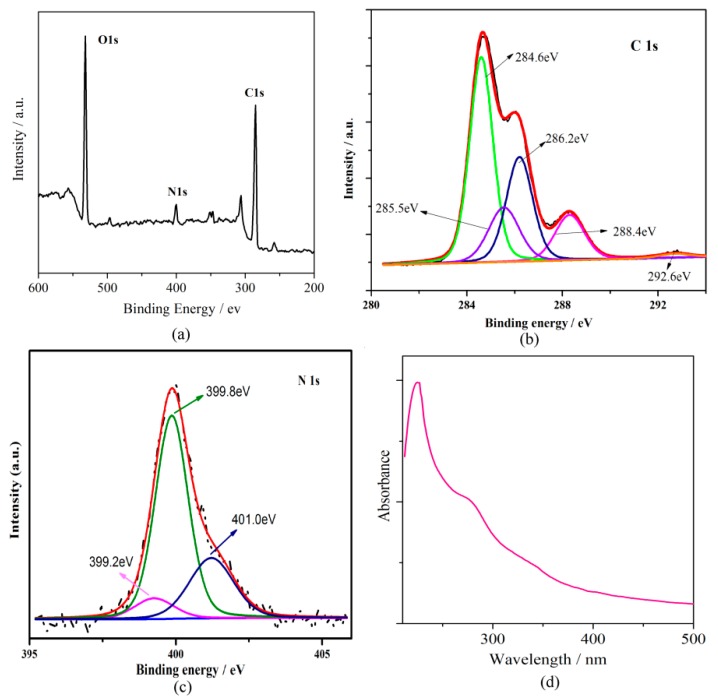
(**a**) XPS spectrum; (**b**) High-resolution C1s XPS spectra; (**c**) High-resolution N1s XPS spectra and(d) UV-Vis absorption spectrum of CDs.

FTIR and XPS plots were determined to investigate the chemical bonds and the compositions of the CDs. Figure 1d shows the O–H and the N–H stretching vibrations in the 3000–3700 cm^−1^ regions as well as the vibrations of C=O, C–N, and C–O at 1641, 1396, and 1095 cm^−1^, indicating the existence of carboxyl, amine, and hydroxyl groups [21]. In the XPS plot of CDs (Figure 2a), peaks at 285.0, 398.9, and 532.1 eV were respectively corresponding to C1s, N1s, and O1s. Figure 2b,c further ensured the existence of C–N, N–H, C–O/C=O, and C–C/C=C bonds in CDs.

As shown in Figure 2d, the as-synthsized CDs displayed two absorption bands centered at 225 and 278 nm, as well as one weak band around 336 nm, which respectively corresponded to the Π–Π* transition of the aromatic sp^2^ structure and the n–Π* transition of carbonyl [22,23].

As portrayed in Figure S1, CDs demonstrated excitation wavelength-dependent property, which indicated the effects of different sized particles and various surface states distribution, as in most reported CDs [22,23,24,25,26].

The absolute quantum yield of CDs is determined to be 11% with the excitation wavelength of 330 nm, which is comparable with other reported CDs [13].

### 3.2. Characterization of CDs-Tb

In order to detect DPA, CDs-Tb has been synthesized as a ratiometric FL nanoprobe (Scheme 2). In this nanoprobe (Scheme 1), CDs are not only act as ligands to coordinate with Tb^3+^, but also serve as a fluorescence reference, while Tb^3+^ ions act as specific recognition unit and response signal.

**Figure 3:**
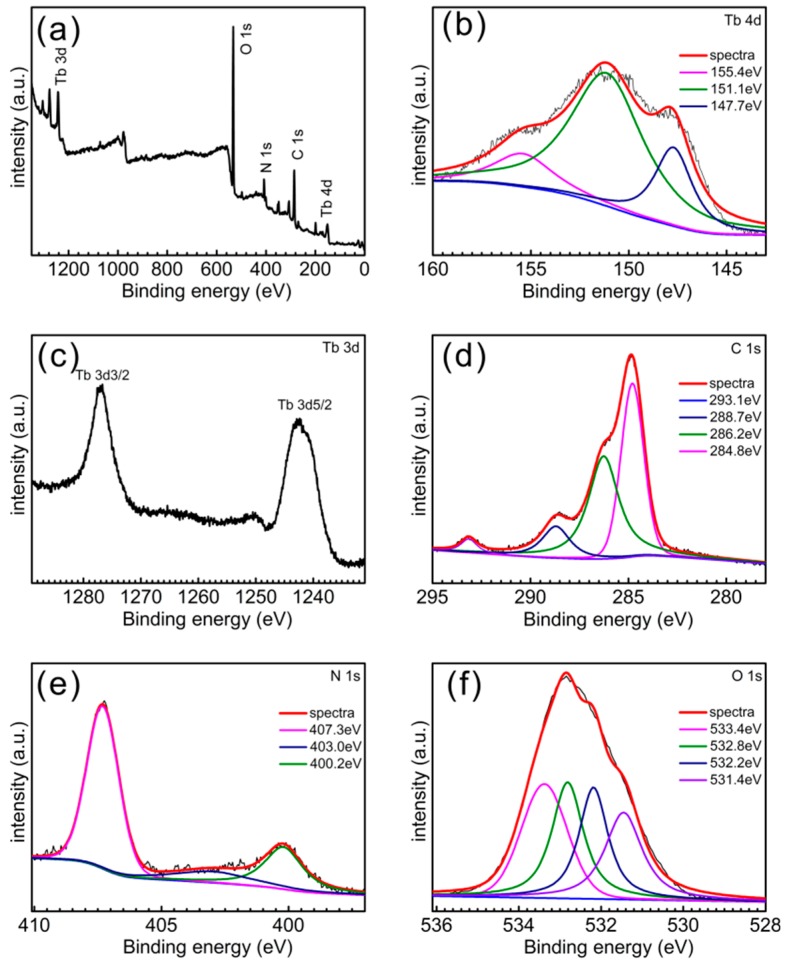
(**a**) XPS spectrum; (**b**) high-resolution Tb 4d XPS spectra; (**c–f**) high-resolution Tb3d, C1s, N1s and O1s XPS spectra of CDs-Tb.

XPS characteration was also carried out to determine the composition of CDs-Tb. Figure 3 clearly indicated the presence of carbon, nitrogen and oxygen in this nanoprobe. Additionally, a weak peak at 151.8 eV (Figure 3a,b) corresponding to Tb 4d is appeared, which confirmed the preparation of the CDs-Tb. Besides, two peaks at 1277.3 and 1242.5 eV corresponded to 3d3/2 and 3d5/2 of Tb^3+^ were observed in the HRXPS spectrum of Tb 3d (Figure 3c).

Optical properties of CDs-Tb including FL emission and UV-vis spectra were investigated. As demonstrated in Figure S2, CDs-Tb showed one broad absorption band around 275 nm. Under the excitation of 265–375 nm, CDs-Tb exhibited excitation-dependent FL property with maximum emission and excitation peaks around 445 and 265 nm, respectively (Figure S3).

### 3.3. Determination of the DPA

**Figure 4:**
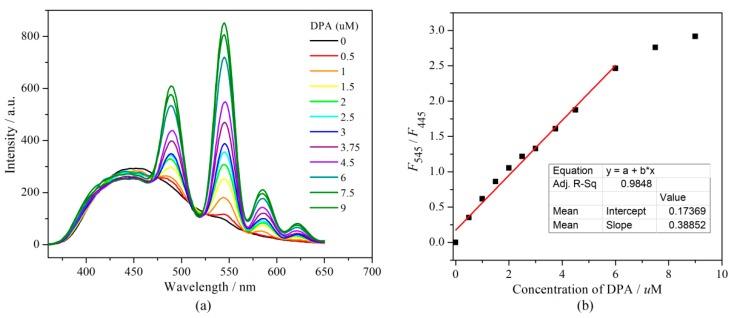
(**a**) Fluorescence (FL) response of CDs-Tb upon the addition of DPA (λ_ex_ = 270 nm); (**b**) ratiometric calibration plot of CDs-Tb (F_545_ /F_445_) and DPA concentration.

The ability of this nanoprobe for detection of DPA was assessed systematically by FL titrations. As delineated in Figure 4a, without the addition of DPA, the emission spectrum of CDs-Tb was dominated by the blue FL emission of CDs centered at 445 nm. Upon addition of various amounts of DPA, the emission intensities of CDs remained nearly unchanged, while the emission intensities of peaks at 489, 545, 586, and 621 nm assigned to Tb^3+^ ions enhanced obviously, owing to effective energy transfer from DPA to Tb^3+^. Thus, the blue FL of the CDs centered at 445 nm served as the reference signal, while the green FL of DPA-sensitized Tb^3+^ served as the response signal in the CDs-Tb nanoprobe. Since the FL emission of Tb^3+^ at 545 nm was the most intense emission peak after addition of DPA, its FL intensity changes were set to quantitatively measure DPA.

Moreover, we investigated the influence of pH on the ratio FL intensity of F_545_/F_445_ in the nanoprobe. As portrayed in Figure S4, the ratio FL intensity remained nearly unchanged within pH range 4.0–8.0 because of the abundant oxygen-containing groups on the surface of CDs and the balance of their protonation and deprotonation. However, this ratio FL intensity remarkably reduced at pH > 9.0 on account of the generation of Tb^3+^ hydroxide [22].

As delineated in Figure 4a, the FL intensity of Tb^3+^ at 545 nm was obviously enhanced upon the addition of DPA. Nearly nine-fold increase was observed when DPA concentration was 9 µM. The ratio FL intensity of F_545_/F_445_ and the DPA concentrations showed a linear relationship with R^2^ = 0.985 in the experimental concentration range 0.5–6 µM (Figure 4b). The following equation was utilized to calculate the detection limit of CDs-Tb toward DPA: the detection limit = 3S_B_/S, where S_B_ was standard error for the blank test, which was determined by 10 continuous scannings of the blank sample, and where S was the slope of the calibration curve. The obtained detection limit was 35.9 nM, which was superior compared to values in previously published literature and significantly lower than the infectious dosage of the spores (60 μM) [4,13,14,16,17,18,19,22].

### 3.4. Mechanism for DPA Detection Using CDs-Tb

**Figure 5:**
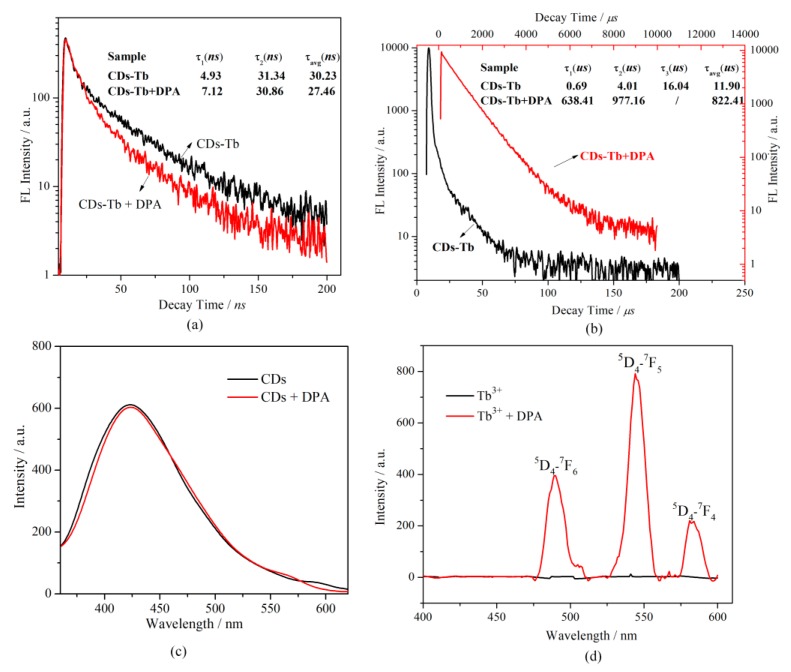
FL decay profiles of emissions at (**a**) 445 nm and (**b**) 545 nm of CDs-Tb and CDs-Tb+DPA (λ_ex_ = 270 nm); FL intensity of (**c**) the CDs (0.5 mg·mL^−1^) and (**d**) the Tb^3+^ ions (1 mM) with and without DPA (60 μM).

We further explored the mechanism for DPA detection. As depicted in Figure 5a,b, the FL lifetime of CDs in the CDs-Tb solution (0.1 mg·mL^−1^) slightly decreased from 30.23 ns to 27.46 ns upon the addition of DPA (80 μM), while that of the green FL of Tb^3+^ in the CDs-Tb significantly increased from 11.90 μs to 822.41 μs. Such phenomenon indicated that DPA could absorb the excitation light and transfer its energy to Tb^3+^ in CDs-Tb effectively, implying the mechanism for DPA detection was attributed to the AETE from DPA to Tb^3+^ [7,8]. As for CDs in CDs-Tb, the reduction of excitation light absorption may have been responsible for its slightly decreased lifetime, which was nearly negligible [13].

Moreover, as demonstrated in Figure 5c,d, the FL intensity of free CDs remained almost unchanged after adding DPA, while the signal of free Tb^3+^ was dramatically enhanced, implying the vital role of Tb^3+^ in DPA detection. As portrayed in Figure 5d, new bands located at 489, 545, and 586 nm were clearly observed, which corresponded to the characteristic emission of Tb^3+^ induced by the effective energy transfer from DPA to Tb^3+^ [21].

### 3.5. Selectivity of DPA Detection

**Figure 6:**
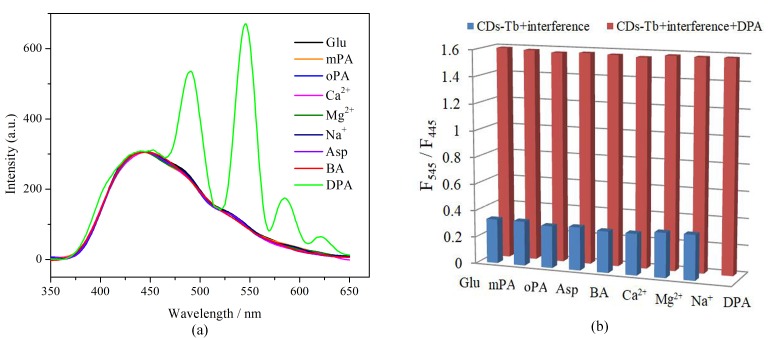
(**a**) Influences of DPA and interfering species on the FL intensity of CDs-Tb. (**b**) Blue bars indicate influences of interfering species (10 μM) on the FL intensity of CDs-Tb and red bars are influences of interfering species and DPA (4 μM) on the FL intensity of CDs-Tb (10 μg·mL^−1^), λ_ex_ = 270 nm.

As another vital parameter for a nanoprobe, selectivity of CDs-Tb was evaluated. The effects of other structurally related species, such as m-phthalic (mPA), o-phthalic (oPA), benzoic (BA), glutamic (Glu), and D-aspartic (Asp) acid, were studied under similar conditions. The effects of various common cellular ions (Ca^2+^, Mg^2+^, Na^+^) were also investigated. Notably, no obvious intensity changes occurred when adding the above interfering species compared to DPA (Figure 6a). Furthermore, the coexistence of the above species did not cause obvious interference for CDs-Tb when sensing DPA (Figure 6b) and allowed for selective determination of DPA.

### 3.6. Analysis in Real Samples

To further evaluate the applicability of this ratiometric nanoprobe toward DPA, some studies were carried out in the lake water sample. Because no DPA was detected in the lake sample by using the CDs-Tb, a recovery measurement was performed. DPA solutions (1, 2, 5 μM) were added into the lake water sample and determined by the CDs-Tb nanoprobe, respectively. With the obtained ratiometric calibration plot of CDs-Tb (Figure 4b), DPA concentrations were determined. Table 1 demonstrated that the relative standard deviation (RSD) of these tests did not exceed 3.79%, while recoveries were within the range from 96.5% to 105.01%. This ratiometric sensing system showed high accuracy and precision toward DPA determination in real samples.

## 4. Conclusions

In summary, a new ratiometric fluorescent nanoprobe, CDs-Tb, was synthesized for DPA detection by grafting Tb^3+^ onto the surface of CDs. The schizochytrium-based CDs with blue fluorescence were facilely synthesized via a hydrothermal process and served as a FL reference and a supporting substrate for coordination with Tb^3+^, while green emission peaks of Tb^3+^ served as the fluorescence response signal owing to the AETE. This nanoprobe displays high selectivity and sensitivity for DPA detection and can realize the monitoring of DPA in lake water samples.

## Figures and Tables

**Table 1:** Detection of DPA in the lake water sample. pH: 7.6; *λ*_ex_: 270 nm; *c*_CDs-Tb_: 10 μg·mL^−1^.

Sample	Add DPA (μM)	Found ^1^ (μM)	Recovery ^2^ (%)	RSD (%)
	1.0	1.05	105.01	3.05
Lake water	2.0	1.93	96.50	2.65
	5.0	5.22	103.26	3.79

^1^ Average of three repeated detections. ^2^ Recovery (%) = (*c*_found_/*c*_added_) × 100. RSD: relative standard deviation.

## Supplementary Materials

The following are available online at https://www.mdpi.com/2079-4991/9/9/1234/s1, Table S1. Comparison of representative fluorescence probes for measuring DPA, Figure S1: FL emission spectra of CDs under different excitation wavelength (*λ*_ex_ = 265–395 nm), Figure S2: UV-Vis absorption of CDs-Tb, Figure S3: FL emission spectra of CDs-Tb under different excitation wavelength (*λ*_ex_ = 265–375 nm), Figure S4: Influence of pH on the ratio FL intensity *F*_545_ /*F*_445_ of CDs-Tb upon addition of 5 μM DPA, *λ*_ex_ = 270 nm.
