# Supplementary material for: Terbium Functionalized Schizochytrium-Derived Carbon Dots for Ratiometric Fluorescence Determination of the Anthrax Biomarker
Source: Nanomaterials (Basel). 2019 Aug 30;9(9):1234. doi: 10.3390/nano9091234 (PMC6780622; doi:10.3390/nano9091234)
Supplement: Supplementary file 1 [file nanomaterials-09-01234-s001.pdf]

Article

Terbium Functionalized Schizochytrium-Derived Carbon Dots for Ratiometric Fluorescence Determination of the Anthrax Biomarker

Lina Zhang [1], Zhanwei Wang [1], Jingbo Zhang [1], Changliang Shi [1,*], Xiaoli Sun [2], Dan Zhao [1] and Baozhong Liu [1,*]

[1] College of Chemistry and Chemical Engineering, Henan Polytechnic University, Jiaozuo 454003, China
[2] Chemical Laboratory, Medical Instrument Testing Institute of Henan province, Zhengzhou 450018, China
* Correspondence: shichangliang@hpu.edu.cn (C.S.); bzliu@hpu.edu.cn (B.L.)

Received: 12 August 2019; Accepted: 27 August 2019; Published: date

Table S1. Comparison of representative fluorescence probes for measuring DPA.

Fluorescence Probes	Linear Range (μM)	Detection Limit (nM)	Reference
Eu(III) functionalized silicon QDs	0–34	1020	[1]
Eu(III)-doped carbon dots	0.005–0.7	5	[2]
Single-walled carbon nanotube-Tb	/	1	[3]
CDs-Cu^{2+} systems	0.25–20	79	[4]
Tb/Eu@bio-MOF	0.05–134	34	[5]
PVA film	0.1–50	100	[6]
Tb-silica NPs	/	56.6	[7]
CDs-Tb	0.5–6	35.9	This work

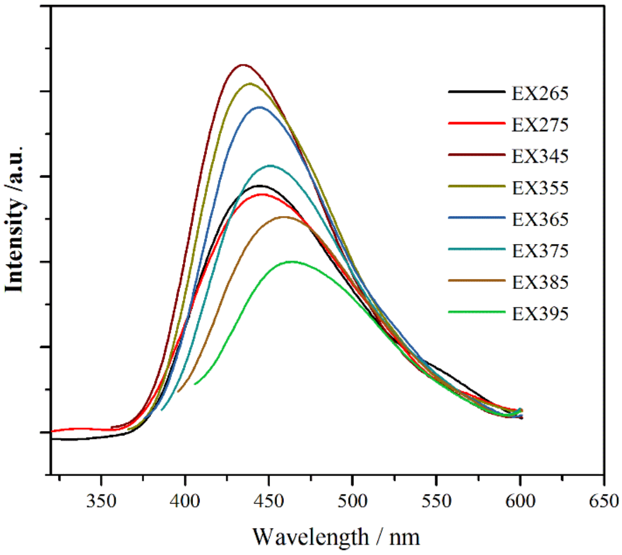

Figure S1. FL emission spectra of CDs under different excitation wavelength (λ_{ex} = 265~395 nm).

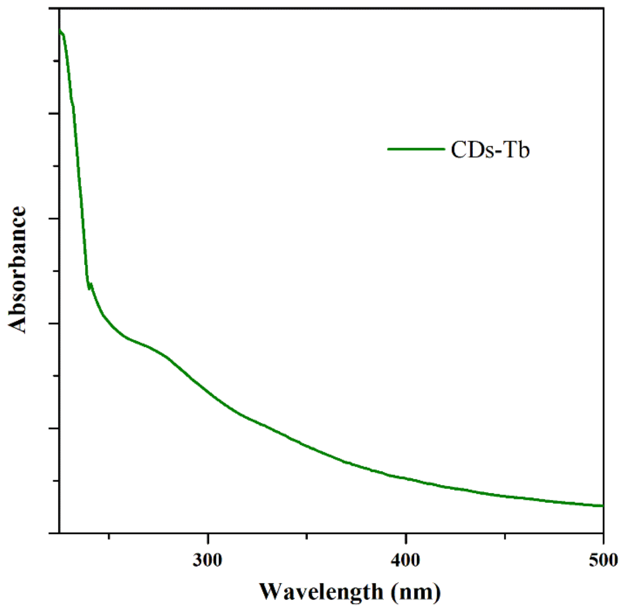

Figure S2. UV-Vis absorption of CDs-Tb.

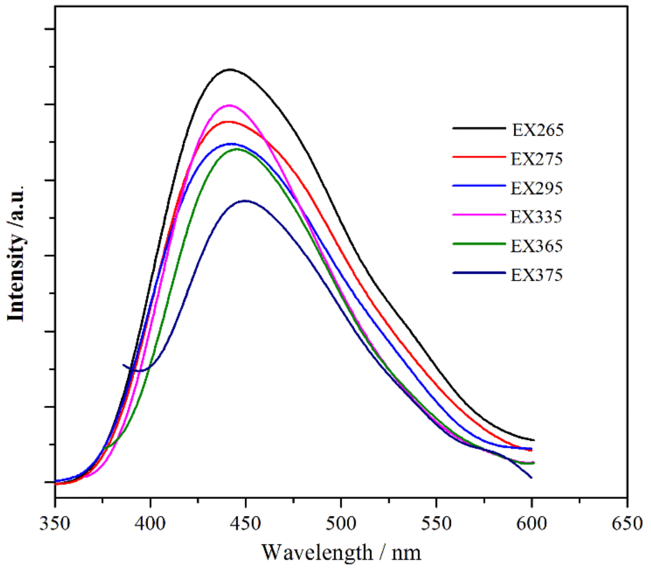

Figure S3. FL emission spectra of CDs-Tb under different excitation wavelength (λ_{ex} = 265~375 nm).

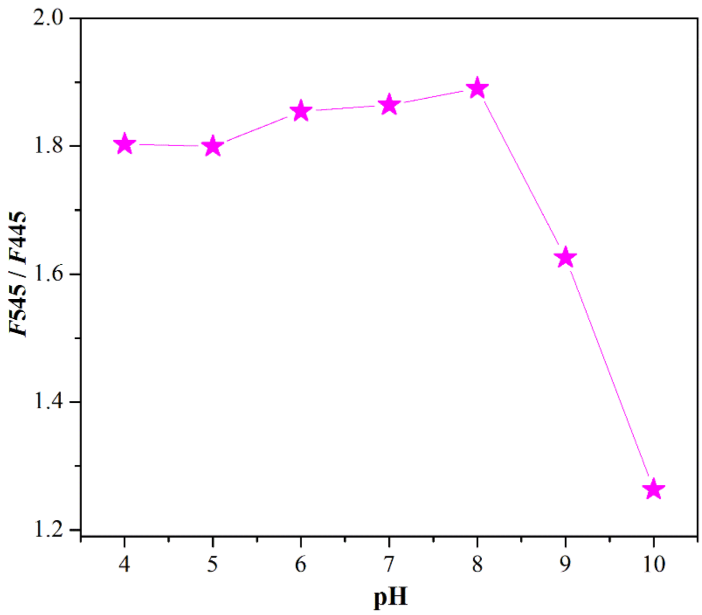

Figure S4. Influence of pH on the ratio FL intensity F_{545}/F_{445} of CDs-Tb upon addition of 5 μM DPA, λ_{ex} = 270 nm.

References

1. Zhou, Z.; Gu, J.P.; Chen, Y.Z.; Zhang, X.X.; Wu, H.X.; Qiao, X.G. Europium functionalized silicon quantum dots nanomaterials for ratiometric fluorescence detection of bacillus anthrax biomarker. *Spectrochim. Acta Part A.* **2019**, *212*, 88–93.
2. Rong, M.C.; Deng, X.Z.; Chi, S.; Huang, L.Z.; Zhou, Y.B.; Shen, Y.N.; Chen, X. Ratiometric fluorometric determination of the anthrax biomarker 2,6-dipicolinic acid by using europium(III)-doped carbon dots in a test stripe. *Microchim. Acta* **2018**, *185*, 201–210.
3. Tian, C.; Wang, Q.; Zhang, C. Optical and electrochemical responses of an anthrax biomarker based on single-walled carbon nanotubes covalently loaded with terbium complexes. *Chem. Commun*. **2011**, *47*, 12521–12523.
4. Li, P.J.; Ang, A.N.; Feng, H.T.; Li, S.F.Y. Rapid detection of an anthrax biomarker based on the recovered fluorescence of carbon dot-Cu(II) system. *J. Mater. Chem. C* **2017**, *5*, 6962–6972.
5. Zhang, Y.H.; Li, B.; Ma, H.P.; Zhang, L.M.; Zheng, Y.X. Rapid and facil retiometric detection of an anthrax biomarker by regulating energy transfer process in bio-metal-organic framework. *Biosens. Bioelectron*. **2016**, *85*, 287–293.
6. Ma, B.L.; Zeng, F.; Zheng, F.Y.; Wu, S.Z. Fluorescent detection of anthrax biomarker based on PVA film. *Analyst* **2011**, *136*, 3649–3655.
7. Taylor, K.M.L.; Lin, W.B. Hybrid silica nanoparticles for luminescent spore detection. *J. Mater. Chem.* **2009**, *19*, 6418–6422.
